# A pragmatic approach to effective anatomy teaching and learning to medical students: a ten-year experience using evidence-based principles

**DOI:** 10.15694/mep.2021.000147.1

**Published:** 2021-05-26

**Authors:** Thomas Goubar, Jonathan Larach, Jake Hindmarch, Suzannah Bownes, Sankar Sinha

**Affiliations:** 1 The University of Notre Dame Australia

**Keywords:** Anatomy, Anatomy teaching, Medical Education, Online learning, Near-Peer Teaching, Team-based learning.

## Abstract

This article was migrated. The article was marked as recommended.

Teaching and learning of anatomy for medical students have been extensively studied. However, we believe that a ‘gold-standard’ of an anatomy teaching and learning model is difficult to establish as every educational institution faces unique. For the past ten years at the University of Notre Dame Australia, School of Medicine Sydney, the anatomy faculty has implemented evidence-based teaching strategies adopted from medical schools around the world and supported by timely student feedback to deliver cost-effective and sustainable anatomy learning. Student evaluations of this program have been positive and associated with improved summative anatomy results. This article describes ten principles pursued by our faculty, which we hope will help others in restructuring or enhancing their anatomy teaching and learning program.

## Introduction

The value of anatomy knowledge as a cornerstone of clinical practice has been highlighted in numerous publications with many useful teaching innovations designed for improved student learning ( Louw, Eizenberg, and Carmichael, 2009; Yammine, 2014; Losco *et al.,* 2017; Vertemati *et al.,* 2018; Singh *et al.*, 2019; Zill, 2020; Baker, 2021). However, the ideal model of best practice anatomy teaching and learning remains an elusive goal under the current climate of an overloaded medical curriculum, limited financial resources, and insufficient anatomy teachers ( Drake *et al.*, 2009; Trautman *,* McAndrew and Craig, 2019). Some studies indicate there may be a perception among senior clinicians about the inadequate level of anatomy knowledge of the current junior doctors ( O–Keeffe *,* Davy and Barry, 2019). In addition, junior doctors have been noted to express concern about their level of anatomy knowledge ( Turney, 2007; Farey *et al.*, 2018; Tayyem *et al.*, 2019). Such issues continue to inspire anatomy educators in their search for better anatomical teaching and learning practices. The present article describes how the anatomy faculty at the University of Notre Dame Australia, School of Medicine Sydney (UNDA SOMS), has improved their anatomy learning strategy over the past decade with good outcomes even during the recent unprecedented Covid-19 crisis.

The adoption of modern teaching methods in recent years, including the utilisation of online resources and self-directed learning, has broadened the scope of anatomy education beyond traditional didactic teaching ( Mathiowetz, Yu and Quake-Rapp, 2016; Singh *et al.*, 2019). There is now good evidence to suggest that teaching using integrated technology not only supplements didactic teaching methods but encourages self-directed learning ( Estai and Bunt, 2016; Atta and Al Qahtani, 2018). This teaching must necessarily be tailored to suit the needs of the individual medical schools, driven by a combination of available resources and context-specific conditions.

Due to heterogeneity seen in anatomy curricula, we believe that there is no available ‘gold-standard’ approach to teaching anatomy to medical students. As such, there is a strong need for research and collaboration between medical schools to develop quality anatomy courses within the constraints of modern medical programs. This article outlines ten principles utilised at the University of Notre Dame Australia, School of Medicine, Sydney (UNDA SOMS) to enhance the teaching and learning experience of anatomy ( Table 1). These ten principles are based on ten years of experience in teaching anatomy to medical students. Furthermore, we found our approach was practical when transitioning to the online environment in response to the COVID-19 pandemic. The pedagogical principles that have been implemented at our medical school have their foundation based on evidence gathered from current research findings and recommendations. We hope that this article can positively contribute to the global community of anatomy educators as a practical, cost-effective, and sustainable resource.

**Table 1.** Ten evidence-based principles for pragmatic and effective anatomy teaching and learning.

1. Pragmatism principle: Adopt evidence-based models to suit the constraints of your institution 2. Resourcefulness principle: Utilise internal assets (senior students), desire to give back (past students), and treasure troves (retired clinicians). 3. Reduction principle: Reduce cognitive overload 4. Discovery principle: Implement weekly themes to aid inquiry-based learning 5. Relevance principle: Demonstrate clinically relevant surface (living) anatomy during small group tutorials 6. Evaluation principle: Regularly evaluate and seek feedback 7. Tailor-made principle: Design the course with the end-users in mind 8. Reinforcement principle: Reinforce, reinforce, reinforce 9. Transitioning principle: Transition into online learning seamlessly when needed 10. Maintenance principle: Maintain an effective online learning environment

## Context

The program at UNDA SOMS is a graduate-entry, four-year Doctor of Medicine program with students from a diverse range of educational backgrounds and currently, each cohort has approximately 132 students. Our anatomy faculty share certain commonalities with other medical schools, namely (1) difficulty in sourcing quality tutors, (2) a significant number of students (roughly twenty percent) with non-science background, (3) allocation of reduced anatomy teaching hours, and (4) limited funding for both technological resources and teachers ( Lockwood and Roberts, 2007; Yammine, 2014; Estai and Bunt, 2016; Cameron *et al.*, 2019; Trautman *,* McAndrew and Craig, 2019; Williams, Hawkins and Khalil, 2020).

Most of the anatomy teaching is delivered in the first and second year of the course as part of the Basic and Clinical Sciences domain. In addition, several anatomy lectures are delivered throughout the third and fourth years following weekly clinical themes, providing a level of vertical integration with clinical practice. Delivery of anatomy content incorporates several modalities, including lectures, tutorials by near-peer tutors in the first year, team-based learning tutorials (TBL) in the second year, wet lab sessions with prosected cadavers, and integrated anatomy workshops (IAW) covering the gross anatomy, radiological anatomy, and pathological anatomy. Before the restrictions imposed by the COVID-19 pandemic, delivery of the program was entirely face-to-face, and online resources were utilised as supplementary materials for student revision.

## Pragmatism principle: Adopt evidence-based models to suit the constraints of your institution

A common problem faced by many institutions is the rigid and limited time allocated for anatomy teaching within the medical curriculum. Core topics in medical education have rapidly expanded over the previous two decades, and as such, there is a greater burden to cover a range of disciplines ( Drake, 2014; Smith *et al.*, 2016). As a result, many institutions have reduced allocated hours for anatomy, impacting the quality and competency of medical graduates in clinically applied anatomy ( Losco *et al.*, 2017). Our solution to this challenge has been to decrease didactic teaching and instead incorporate a range of modern pedagogical approaches incorporating active learning, such as team-based learning (TBL) and integrated anatomy workshops (IAW). The near-peer tutor (NPT) led sessions to allow content to be delivered by peers who have excelled, making the content relatable, relevant, and palatable to students learning anatomy for the first time ( Evans and Cuffe, 2009; Border *et al.,* 2017). TBL offers a flipped classroom model, the benefit of which is decreased reliance on class time to teach fundamentals, whilst classroom contact hours are reserved to seek clarification on challenging concepts ( Lahoud *et al.*, 2019). IAW offers students the opportunity to visit anatomical concepts within the context of other medical disciplines such as pathology and radiology. These modalities focus on teaching clinically applied anatomical knowledge, which is an essential medical education outcome ( Estai and Bunt, 2016). The goal is to adopt the pragmatic pedagogical approach suitable for your institution’s time and resource constraints.

## Resourcefulness principle: Utilise internal assets (senior students), desire to give back (past students), and treasure troves (retired clinicians)

Similar to many other universities, we face difficulties in sourcing quality anatomy teachers within the financial constraints of a medical school’s anatomy budget. To address this issue, we have secured tutors from three sources. Firstly, we encourage current senior medical students to participate as NPTs; we call these tutors our ‘internal assets’ ( Williams, Hawkins and Khalil, 2020). The literature suggests students taught by NPTs achieve similar learning outcomes as those led by faculty tutors, with the added benefit of being cost-effective ( Yu *et al.*, 2011). Secondly, we call on our recent graduates to teach anatomy - an opportunity to ‘give back’ to their medical school. Finally, we found many retiring or recently retired clinicians as a ‘treasure trove’ of wisdom. Many clinicians have amassed a wealth of knowledge throughout their careers and a desire to teach the next generation providing an excellent teaching resource for clinically applied anatomy.

## Reduction principle: Reduce cognitive overload

A key issue facing any medical student learning anatomy is the vast amount of factual information that requires memorisation. Cognitive overload is a well-known phenomenon experienced by students, which impairs learning ( Young *et al.*, 2014). To combat this, we have prompted our medical students to ask a key question not only to themselves but also to their tutors: “why do I need to learn this anatomical fact?”. There is evidence to show that reducing cognitive overload in multimedia and anatomical models can lead to effective learning ( Mayer and Moreno, 2003; Chan and Cheng, 2011; Mayer, 2020). Our anatomy department has also found the implementation of low-fidelity simulation anatomical models as an effective tool to aid the learning of complex structures such as the anatomy of the inguinal canal ( Hindmarch *et al.*, 2020). As anatomy educators, we tend to remind ourselves that the objective is to develop effective and efficient doctors and not anatomists. Therefore, every piece of anatomical knowledge should accompany examples of a clinical application ( Louw, Eizenberg and Carmichael, 2009).

## Discovery principle: Implement weekly themes to aid inquiry-based learning

Discovery-based learning can be thought of as any situation where target information is not directly given to the learner but through providing material and minimal guidance learners can work through a series of problems to discover the solution ( Alfieri *et al.*, 2011). We have found that by emphasising problem-solving skills and providing a semi-structured approach to teaching, we facilitate the concept of “Inquiry-based learning” ( Thistlethwaite *et al.,* 2012). It has been demonstrated that the effectiveness of anatomy learning is enhanced when the structure and function of human anatomy are linked to the relevant pathology ( Klement, Paulsen and Wineski, 2011; Atta and Al Qahtani, 2018). We teach clinical anatomy to our medical students by utilising case-based learning in which students focus on a specific pathology ‘theme’ every week. Each week, the theme is aligned with the curriculum of clinical sciences and clinical skills. For example, a clinical science week on cardiac pathology would align to a theme focusing on the anatomy of the heart and the relevant surface anatomy used for diagnosing common cardiac murmurs and other common pathologies. By aligning anatomy teaching with clinical medicine, we allow our students to learn both the structure and functional basis of all significant pathologies.

## Relevance principle: Demonstrate clinically relevant surface (living) anatomy during small group tutorials

Thorough knowledge of surface anatomy is essential to practising safe clinical medicine. Understanding surface anatomy is essential when palpating anatomical structures, performing procedures such as a cricothyroidotomy or lumbar puncture and interpreting medical imaging ( Fillmore, Leech and Tunstall, 2020). Our tutors teach surface anatomy in small group tutorials of approximately ten students. It is well established that students are more confident applying knowledge in small group settings than in didactic pedagogy ( Singh *et al.*, 2016). Using markers, tutors will draw on a student volunteer and outline the clinically relevant surface (living) anatomy. Important landmarks covered include relevant vasculature used for venepuncture, lines of pleural reflections and location of lung lobes relevant to auscultation, and the location of the heart border and cardiac valves to ensure students auscultate the correct areas during cardiac examinations. This approach to surface anatomy learning has been implemented in other institutions to great success, whereby students expressed feeling more confident in physical examination and professionalism ( Chinnah, De Bere and Collett, 2011).

## Evaluation principle: Regularly evaluate and seek feedback

We believe that quality improvement processes help create positive learning environments, which has also been demonstrated in the literature ( Wong and Headrick, 2020). For feedback to be meaningful to academic staff and students, it should be administered promptly and timely, and there must be evidence that the department is acting on the input. This fosters a robust educational alliance with the student cohort and allows for collaboration and flexibility in how anatomy learning is delivered.

During the COVID-19 pandemic, student feedback was integral to the process of implementing the online course ( Kelsey *et al.*, 2020). UNDA SOMS rapidly implemented changes to accommodate new online learning environments. Hence, it was imperative for our department to regularly monitor specific outcomes to ensure teaching and learning quality.

Quality assurance should incorporate both qualitative and quantitative markers of success. Regular surveys can determine student satisfaction and engagement, which will facilitate the direction of resources to the most effective teaching and learning strategies for your student body. An effective way to obtain quantitative data was in the UNDA SOMS weekly pre and post-class quizzes. This offers a two-pronged approach to quality assurance, in that quantitative assessment can be conducted in the quizzes, and qualitative measures of satisfaction and engagement can be assessed.

## Tailor-made principle: Design the course with end-users in mind

The goal of most anatomy programs will be to increase the students’ confidence in applying anatomical knowledge in clinical situations. A simple way we have implemented quality assurance measures is to employ the Kirkpatrick model of learning evaluation ( Galloway, 2005). The model describes four levels of assessment for a comprehensive analysis of a training program: Reaction, Learning, Behaviour and Results.

Firstly, Reaction, which measures students’ response to training (i.e., the level of satisfaction and engagement via feedback surveys) will ensure that students are engaged with content delivery. Learning analyses the level of knowledge attained from the teaching (i.e., formative quizzes). Behaviour determines whether students are then applying their acquired knowledge in new situations (e.g., has there been a behaviour change), which can be achieved via the implementation of practical labs and clinical questions. Finally, Results look at whether the material had the intended impact on the students (e.g., outcome measures by the educators). We describe this program design as ‘tailor-made’ in light of teaching methods being customised to the necessary, practical, uses of anatomical knowledge for junior doctors. Adopting the strategy described above under the evaluation principle, we have fulfilled the Kirkpatrick model criteria by being mindful of our students’ future needs.

## Reinforcement principle: Reinforce, reinforce, reinforce

Much of the modern literature around memory emphasises the importance of spaced repetition, that is, the practice of repeatedly retrieving information from memory at increasing time intervals ( Karpicke and Bauernschmidt, 2011). At our medical school, we delivered the same topic in the sequence of lecture, practical, and then tutorial. In practice, this means that if we have five hours per week as dedicated anatomy teaching time, we commit one of those five hours to revise concepts from the previous week during the tutorial and practical classes. Furthermore, and true to the spirit of spaced repetition, we conduct revision lectures, tutorials, and practicals closer to the students’ exams. In addition, we implement ‘vertical integration’, a technique whereby we revisit content during the later years of the medical course aligned with clinical teaching themes ( Eisenstein *et al.*, 2014). We have received, and continue to receive, positive feedback from our students about this approach. This is correlated with a decreasing number of students scoring less than fifty percent marks in their exams and an increasing number of students scoring greater than seventy-five percent marks in their summative exams in anatomy over the past ten years.

## Transitioning principle: Transition into online learning seamlessly

The growing supply of online anatomy education resources and the recent COVID-19 pandemic has driven the UNDA SOMS anatomy curriculum online, much like many other institutions ( Gaur *et al.*, 2020; Harmon *et al.,* 2020; Wang, Xie, Wang and Wu, 2020). Thanks to the existing learning framework and teaching modalities, the anatomy faculty could transition to online teaching and learning quickly, seamlessly, and without interruption. We have a few key recommendations to share based on the experience of this transition.

Firstly, we recommend having a robust learning management system in place before making the transition. At the UNDA SOMS, the use of the Blackboard (Blackboard LLC, Washington, DC) allows administrators to publish resources, track online classes and create an effective platform that can form the basis for online learning. Secondly, through Zoom (Zoom Video Communications, San Jose, CA), the anatomy lectures and group tutorials were delivered synchronously in an online format. Zoom allows educators to share content and engage students through group discussions and polling questions. Furthermore, Zoom enables educators to track student attendance and engagement through chat, in-class polling questions, and back-end features such as usage reports. Next, we suggest providing students access to authentic online anatomy education resources, such as Acland’s Video Atlas of Human Anatomy, an online resource with high-quality videos of prosected human cadavers ( Phinney, 2020). Finally, it is essential to ensure that online resources are readily accessible to all students to promote equity.

## Maintenance principle: Maintain an effective online learning environment

Once the transition to online learning has been made, it is essential to maintain this environment to minimise disruptions to student learning. This means ensuring that your university is equipped to deal with internet server outages and other IT troubleshooting, which is vital to online learning success. Detailed instructions of alternative arrangements should be made available for students who don’t have stable internet access. These may include such things as downloadable content for offline use, printouts, and access to university IT resources. Where possible, students should have access to university computers to ensure that the online learning environment is inclusive. We suggest creating an online support group where students can interact with each other and discuss difficult concepts. This year we made a Facebook (Facebook, Menlo Park, CA) group; however, other platforms such as WhatsApp (WhatsApp Inc, Mountain View, CA) or Google Drive (Google LLC, Mountain View, CA) would also be sufficient. These must be moderated and adhere to institutional policies on social media usage. In our teaching, we have found that by re-formatting lectures to include one or two concepts or themes for a short time interspersed with a mini-quiz, significantly enhanced students’ engagement and created an effective online learning environment.

## Conclusion

Delivering an anatomy course that is clinically relevant for medical students is complex. There is a wealth of literature available to anatomy educators involved in teaching medical students. However, this wealth of information has not translated into one ‘gold-standard’ model of anatomy teaching. We propose that a universally applicable ‘gold-standard’ model is difficult to achieve as different educational institutions will each face unique issues and constraints. When establishing or improving anatomy courses institutions should hybridise to suit their own needs and context. These ten principles from The University of Notre Dame, School of Medicine Sydney, have been developed and tested over ten years and are based on research, student feedback, and our medical faculty’s input. We hope our experiences can help other educational facilities enhance their pedagogical process and ultimately reach their ‘gold-standard’ in teaching anatomy to their medical students.

## Take Home Messages

• There is no ‘gold-standard’ model for effective anatomy teaching and learning, a course must be adapted to the constraints of the institution.
• COVID-19 offered an opportunity to trial online anatomy teaching and learning which can be seamless and effective.
• Flexibility in course delivery is essential.
• Feedback should be prompt and there must be evidence that the faculty is acting on this.
• Overall a teaching and learning program needs to be pragmatic, cost-effective, and evidence-based to be sustainable.

## Notes On Contributors

**Dr Thomas Goubar** is a lecturer at the University of Notre Dame Australia, School of Medicine Sydney and doctor at Wagga Wagga Base Hospital. He is currently undertaking a PhD in the role of metabolic surgery in obese patients with heart failure. His research interests include medical education, metabolic surgery and heart failure. In 2020 Thomas was awarded the medical educator of the year award at UNDA SOMS for his contributions to anatomy education. ORCiD ID: https://orcid.org/0000-0002-5873-1610

**Dr Jonathan Larach** has completed a B. Science (Hons I) and a Doctor of Medicine degree. He has been involved in primary, epidemiological and clinical research. His current major interests involve medical education and pain medicine. He has been involved in research spanning across numerous fields including: geriatrics, obstetrics and gynaecology, anaesthetics, gastroenterology and medical education. ORCiD: https://orcid.org/0000-0003-3429-2868

**Dr Jake Hindmarch** holds a Doctor of Medicine and a Bachelor of Biomedical Science which included preliminary training in research methods. His areas of interest include anatomy education and general surgery, having recently published his findings in the ANZ journal of surgery. ORCiD: https://orcid.org/0000-0002-5706-6903

**Suzannah Bownes** is a final year medical student at the University of Notre Dame Australia, School of Medicine, Sydney. She is currently finalising her clinical studies at the Wagga Wagga Clinical School. Her research interests include utilising epidemiological approaches to study primary health issues, as well as medical education. In 2020, she was awarded the Young Investigator Award in General Medicine at the Riverina Medical and Surgical Symposium. ORCiD: https://orcid.org/0000-0002-6456-701X

**Professor Sankar Sinha** has an extensive background in medical education and received the Medal of the Order of Australia for his outstanding contributions to medical education and wound care in 2005 and University of Tasmania Vice-Chancellor’s Medal for Sustained Commitment to Teaching Excellence in 2019. He was a general surgeon practicing in Australia, India, Zambia, and Papua New Guinea. He is currently a Professor in Surgery at the University of Tasmania and Professor and Head of Anatomy at the University of Notre Dame Australia Sydney. ORCiD: https://orcid.org/0000-0002-9676-4480
