# Supplementary material for: A pragmatic approach to effective anatomy teaching and learning to medical students: a ten-year experience using evidence-based principles
Source: MedEdPublish (2016). 2021 May 26;10:147. [Version 1] doi: 10.15694/mep.2021.000147.1 (PMC10939563; doi:10.15694/mep.2021.000147.1)
Supplement: Peer review history

# Reviewer report: Reviewer response for version 1

**DOI:** 10.21956/mep.19106.r27551

**Published:** 2021-06-02

**Authors:** John McLachlan

**Affiliations:** 1 UCLan

This review has been migrated. The reviewer awarded 4 stars out of 5 This article describes experiences within an individual medical school relating to the teaching of anatomy. It attempts to distil these experiences into 10 principles which it describes as evidence-based. The 10 principles are shown and summarized in Table 1 of the paper. The abstract indicates that these principles are associated with improved summative anatomy results.Despite the commendation of evidence-based approaches, this improved performance in assessment outcome is only very briefly and inadequately addressed during the paper itself. Since the authors promote the value of adhering to evidence, it would have been better if they had presented evidence of improvements in anatomy assessment scores following the introduction of the new principles in a constructed manner, either in this paper, or in a companion paper which they could reference.Others looking at the same elements of information may have categorized them in a different way. For instance, assessment appears informally in several of the different principles but could easily be regarded as an essential principle in its own right. Reference could usefully have been made here to programmatic assessment. And the last two principles ‘transition into online learning seamlessly’ and ‘maintain an effective online learning environment’ could readily have been incorporated into one principle.Other principles are both uncontroversial and welcome. The authors promote the use of spaced repetition and regular testing (through the form of Teams Based Learning), Others might disagree with particular aspects within the 10 articulated principles. For instance, authors commend the use of senior students in teaching anatomy, which certainly accords with the experience elsewhere, but also promote the use of retired clinicians and surgeons to teach anatomy which in my experience can cause problems. Retired clinicians may teach in an old-fashioned manner and to a level of anatomical knowledge which is not part of the current curriculum. In addition they may be uncomfortable with digital approaches to anatomy such as three-dimensional reconstruction and 3d printing, and with the use of digital platforms in teaching.In the opinion of this reviewer, the essential topics in producing the undifferentiated junior doctor, the ‘iatroblast’ as it were, are that they have a good understanding of living anatomy and medical imaging since it will be these that they encounter first in the clinical setting. The level of anatomical knowledge needed for surgery, other than minor surgery, is more appropriately regarded as a postgraduate activity. Incorporation of postgraduate teaching in the same environment as undergraduate teaching can lead to valuable cross-fertilization between the two, but it is not the purpose of an undergraduate curriculum to teach surgically specific anatomy in an undergraduate setting. An attempt to write a comprehensive synthesis or systematic review of evidence-based approaches to anatomical education in a medical context would be a major undertaking. As the authors indicate, therefore, this article represents a discussion piece based on their own views and experiences, even if informed by evidence. While it will be clear that I personally do not entirely agree with the structuring of the article, and indeed disagree over some of the particular methodologies used, nonetheless I consider it as potentially an interesting and valuable stimulus to discussion in anatomy teaching units and medical schools elsewhere, and on that basis, I am happy to rate it as ‘four star’ in accordance with the guidance provided to reviewers.

Reviewer Expertise:

NA

No decision status is available

# Reviewer report: Reviewer response for version 1

**DOI:** 10.21956/mep.19106.r27550

**Published:** 2021-06-02

**Authors:** Balakrishnan Nair

**Affiliations:** 1 Centre for Medical Professional Development and University of Newcastle

This review has been migrated. The reviewer awarded 4 stars out of 5 Most medical schools are struggling to teach anatomy . So this evidence based pragmatic approach is very useful for all schools. Their approach to teaching, using senior peers, recent graduates and "treasure troves" is very practical . It will be interesting to see the outcome of this teaching based on the Kirkpatrick Model

Reviewer Expertise:

NA

No decision status is available
